# Ideal pin length and interval in tension band wiring using ring pins for transverse olecranon fractures: a biomechanical study

**DOI:** 10.1186/s12891-025-08828-0

**Published:** 2025-06-06

**Authors:** Seung Hoo Lee, Young Ho Lee

**Affiliations:** 1 Department of Orthopaedic Surgery, Chungnam National University Sejong Hospital, Chungnam National University College of Medicine, Sejong, South Korea; 2 Department of Orthopaedic Surgery, Seoul National University Hospital, Seoul National University College of Medicine, 101 Daehak-ro, Jongno-gu, Seoul, 03080 South Korea

**Keywords:** Ring pin, Olecranon fracture, Tension band wiring, Eyelet pin

## Abstract

**Background:**

Several clinical and biomechanical studies on tension band wiring (TBW) using a ring-pin system have been conducted, but no consensus has been reached on the ideal surgical technique. In this study, we aimed to determine the ideal interval and length of ring pins for the treatment of transverse olecranon fractures using TBW with a ring-pin system.

**Methods:**

A biomechanical study was performed using 32 fourth-generation composite ulnae and a ring-pin system specially designed for TBW. Four groups of eight sawbones were created based on the interval and length of the ring pins. A cyclic loading test was performed to measure stability during the active range of motion exercises. A load-to-failure test measured the maximal load until fixation loss.

**Results:**

All groups were stable, with a micromotion of < 1.0 mm, except for Group 3 (length: 50 mm, interval: 10 mm) during the cyclic loading test. The mean micromotion and displacement of Group 3 were significantly higher than those of Groups 2 and 4 (length: 90 mm, interval: 10 mm). The maximal load to failure in Group 3 was significantly lower than that of Groups 2 and 4.

**Conclusion:**

Inserting two ring pins in parallel at a 10-mm interval with a length of ≥ 70 mm for TBW in transverse olecranon fractures is recommended. Further widening of the pin interval provides no biomechanical benefit and may result in technical difficulties owing to the anatomical features of the ulna; in summary, 50-mm ring pins show significantly lower mechanical strength.

## Background

Olecranon fractures are relatively common injuries, representing approximately 10% of all fractures in the upper extremity. Among them, simple transverse olecranon fractures are the most prevalent, accounting for up to 85% of all cases [1, 2]. The primary goal in treating olecranon fractures is to restore the integrity of the articular surface and ensure adequate stability [1]. This approach aims to facilitate early mobilization and prevent the development of elbow stiffness [1]. Although plate fixation is recommended for complex fractures with significant fragmentation [1], simple transverse olecranon fractures are often treated using tension band wiring (TBW), which is an established fixation method [3].

Although TBW is widely accepted, it can lead to complications, such as skin irritation and K-wire backing out [4]. To address these issues, the AO-modified technique recommends passing a K-wire into the anterior ulnar cortex to reduce wire migration [4]. However, this also increases the risk of complications such as damage to the radial nerve, penetration of the articular surface, and impingement on the proximal radius and biceps tendon, resulting in limited forearm rotation and pain [5].

**Fig. 1:**
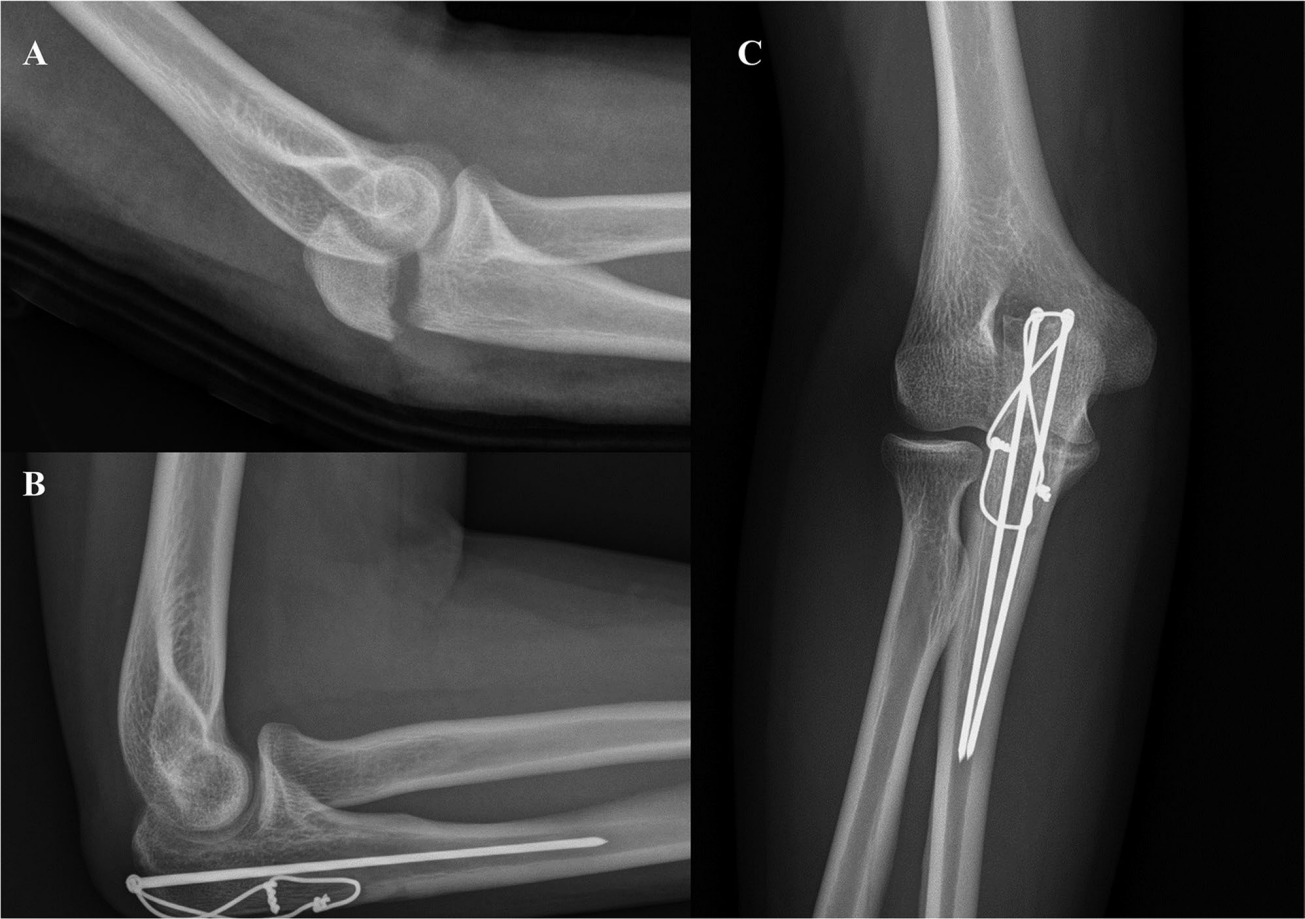
Transverse olecranon fracture (**A**), which was treated by tension band wiring using ring pins (**B** and **C**). Ring pins were inserted along the intramedullary space of the ulna

To avoid these complications, we treated olecranon fractures using TBW with ring pins (eyelet pins) (Fig. 1A, B and C), and several studies have reported positive clinical outcomes [6, 7]. The ring pin was designed to be inserted at the intramedullary position instead of engaging the anterior cortex of the ulna (Fig. 1A, B and C). Despite several studies on the clinical outcomes and biomechanical properties of TBW with a ring-pin system [6–9], there is no consensus on the optimal surgical technique for TBW with ring pins, such as the length of the pin and interval between pins, resulting in variations in the surgical technique. We have frequently received inquiries about the ideal pin length and interval between pins when we presented our clinical results at a domestic conference. However, because of a lack of biomechanical studies on this topic, we recognized the need to investigate the optimal interval and length of ring pins used for TBW with a ring-pin system. Therefore, in this study, we aimed to determine the biomechanically ideal interval and length of ring pins for the treatment of transverse olecranon fractures using TBW.

## Methods

**Fig. 2:**
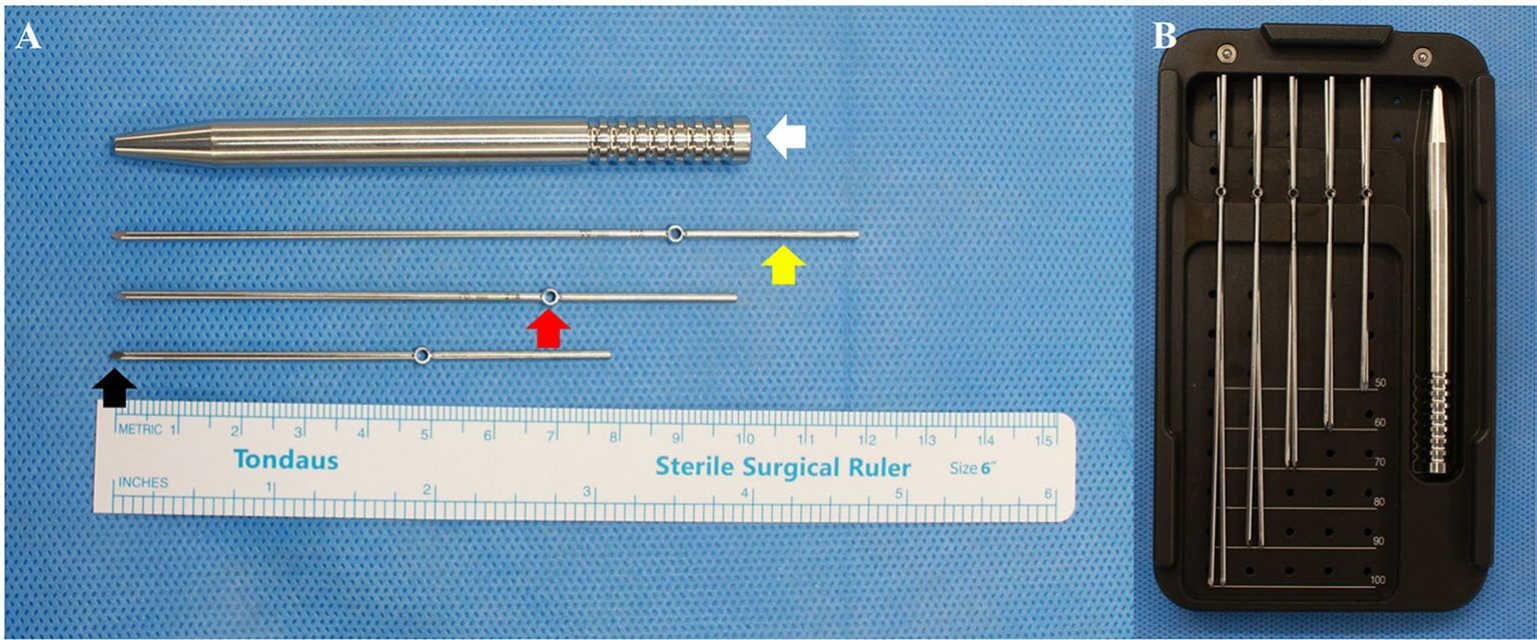
Ring pin set (Jeil Medical, Seoul, Korea). (**A**) It consists of three parts: the proximal tail (yellow arrow) for securing the grip for the power drill, the ring (red arrow) for the passage of the cerclage wire, and the sharp distal end for intramedullary insertion into the ulna (black arrow). A snapper (white arrow) is used for further advancement of the ring pin and removal of the proximal tail (yellow arrow). (**B**) Ring pins are provided in lengths from 50 mm to 100 mm

**Fig. 3:**
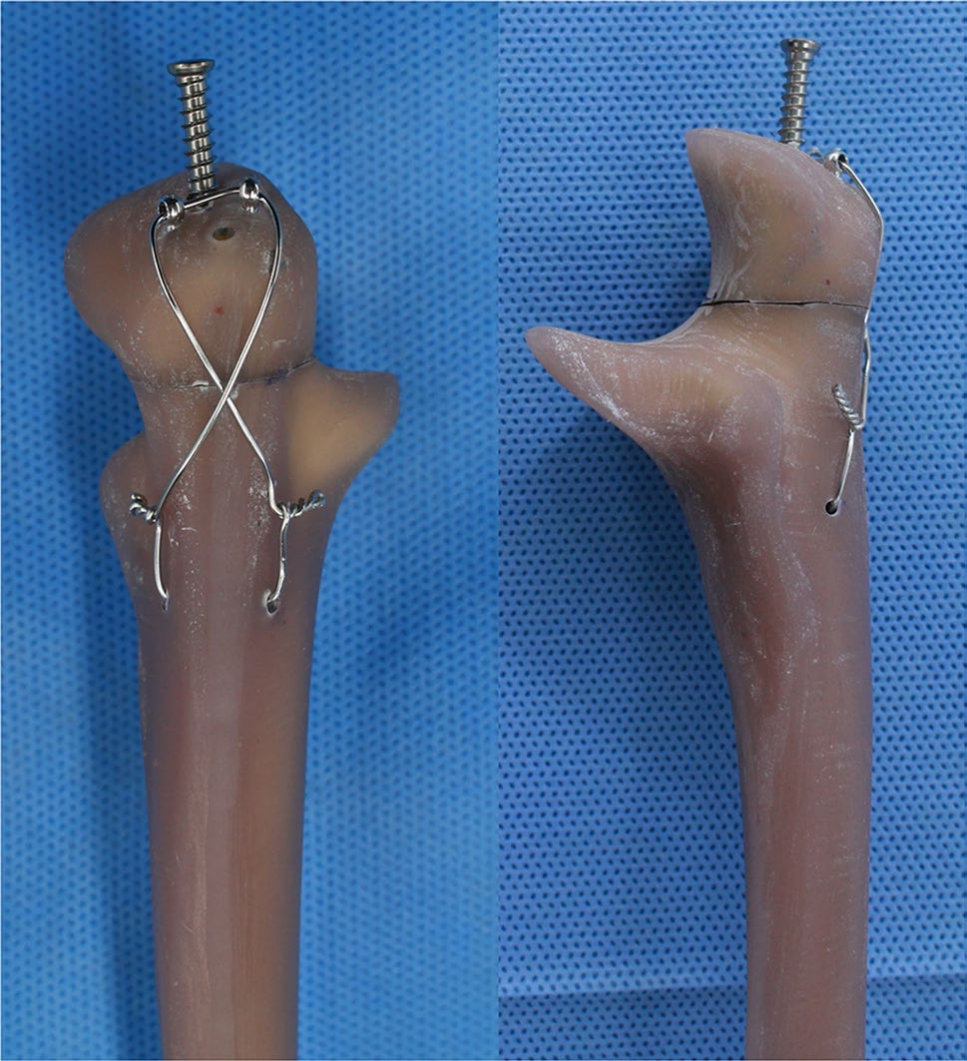
Transverse olecranon fracture model, which was fixed by tension band wiring using ring pins. Bypassing the cerclage wire through the ring, the pin is secured, thus making the proximal migration of the pins unlikely unless the cerclage wire breaks. Consequently, complications associated with pin migration are minimized

This study was approved by the Institutional Review Board of our hospital (No. NON2023-001), and the need for informed consent was waived by the ethics committee because the study did not involve human participants. This biomechanical study used 32 fourth-generation composite ulnae (Sawbones, Pacific Research Laboratories, Vashon, WA, USA), which had very similar structural properties to their cadaveric counterparts with less variability [10, 11]. The ring pin used in this study was a specially designed product for TBW from Jeil Medical (Korea). This ring pin has gained widespread use for TBW to surgically treat fractures such as olecranon and patella fractures. This implant consists of three components: a proximal tail (yellow arrow) designed to secure the grip for the power drill, a ring (red arrow) for the passage of the cerclage wire, and a sharp distal end (black arrow) intended for intramedullary insertion into the ulna (Fig. 2A). A snapper (white arrow) is used for further advancement of the ring pin and removal of the proximal tail (yellow arrow) (Fig. 2A). Ring pins are provided in lengths from 50 mm to 100 mm in 10-mm increments and are 1.6 mm in diameter (Fig. 2B). The pin is secured after the cerclage wire is threaded through the two rings (Fig. 3), making the proximal migration of the pins unlikely unless the cerclage wire breaks; thus, the risk of complications associated with pin migration are minimized (Fig. 3).

### Specimen preparation

An oscillating saw was used for osteotomy at the center of the semilunar notch to create a transverse olecranon fracture (Mayo Type 2A) model [1]. To prevent a reduction clamp slippage, a drill hole (diameter: 2.4 mm) was made 2 cm distal from the osteotomy site. For wire passage, a coronal hole with a 2.0-mm diameter was created in the proximal part of the ulna, 4 cm distal from the osteotomy site, and positioned 5 mm away from the posterior cortex using a 2.0-mm drill. The fracture was then reduced using one or two reduction clamps and maintained by tightening the clamp. To allow for later advancement, ring pins were inserted along the intramedullary cavity of the ulna, protruding 5–10 mm from the bone. A cerclage wire (18 gauge) was threaded through the predrilled coronal hole of the ulna and then through two rings in a figure-of-eight method. Two wire twists were placed on each ulna side and tightened simultaneously. This ensured consistent and balanced tension across the construct (Fig. 3). The protruding pins were impacted using a snapper and hammer until bone contact was established, enabling additional tightening. The tails were subsequently removed using a snapper (Fig. 2A).

**Fig. 4:**
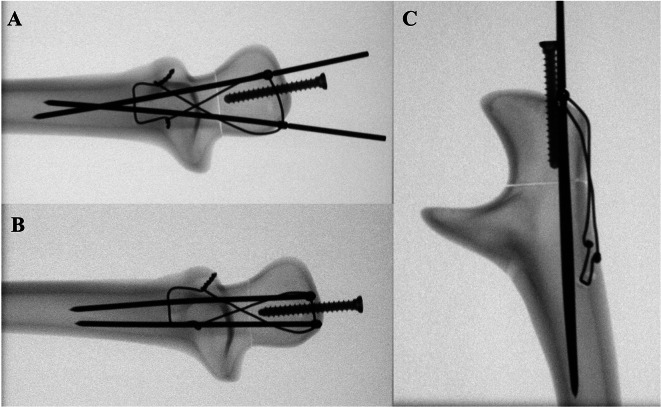
C-arm images of specimens according to the length and interval of ring pins. (**A**) Two 70-mm ring pins were inserted at 20-mm intervals. (**B** and **C**) Two 70-mm ring pins were inserted at 10-mm intervals

Four groups (each consisting of eight sawbones) were created to compare differences according to the interval and length of the ring pins. In Group 1, two 70-mm ring pins were inserted at a 20-mm interval, and they were inserted in a converging direction to avoid penetration of the cortical portion (Fig. 4A). In Group 2, two 70-mm ring pins were inserted in the parallel direction at a 10-mm interval (Fig. 4B and C). Two 50-mm ring pins were inserted in Group 3 and two 90-mm ring pins in Group 4 in the parallel direction at a 10-mm interval. Groups 1 and 2 were compared to analyze the biomechanical differences according to the pin insertion interval, and Groups 2, 3, and 4 were compared to analyze the differences according to pin length.

### Biomechanical testing

**Fig. 5:**
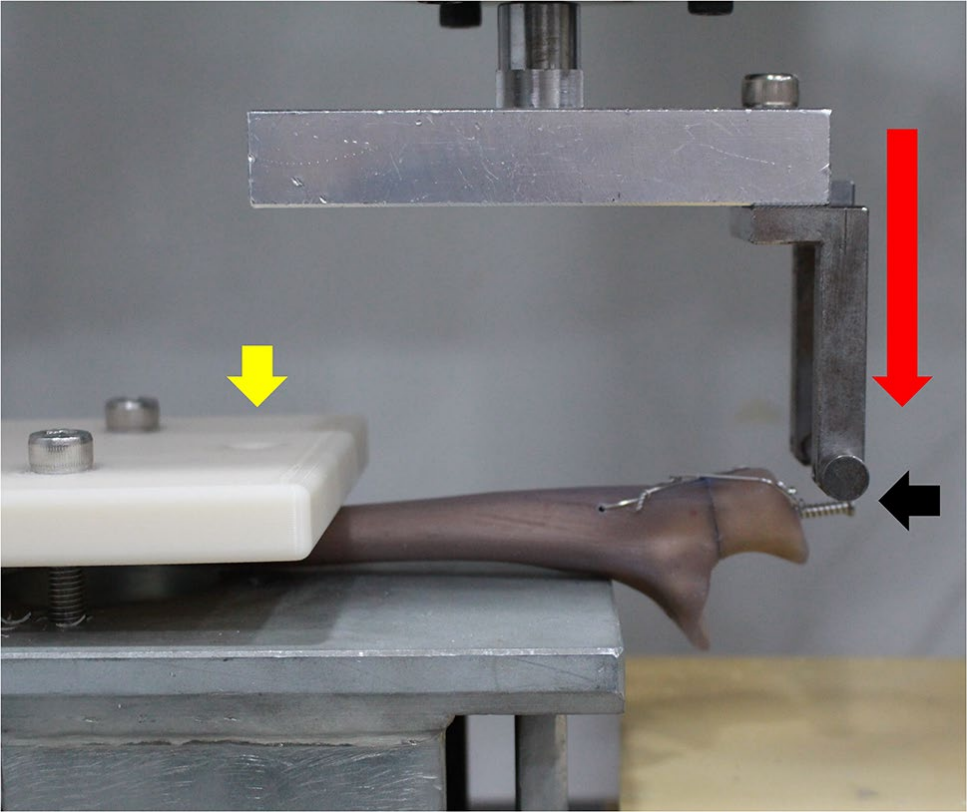
Biomechanical testing setup using an Instron E3000 (Instron Engineering Corporation, Norwood, MA). Specimens were fixed in a positioning apparatus customized for this study (yellow arrow). The load (red arrow) was applied to the load screw (black arrow) in the direction of 90° of the ulnar axis. The load cell’s moving distance was assumed to be an approximation of the micromotion during the test. Fixation loss was defined as an increase of 2 mm or more in the moving distance of the load cell or when a catastrophic failure occurred

The setup of the biomechanical test was adjusted based on the protocols used in previous studies [12]. The mechanical testing machine used in this study was the Instron E3000 (Instron Engineering Corporation, Norwood, MA, USA) (Fig. 5). Specimens were secured at the same point of the ulna in the positioning apparatus customized for this study (yellow arrow) to eliminate errors caused by the experimental conditions (Fig. 5). To eliminate interference caused by the polyester band or steel wire, the load (red arrow) was applied to the load screw (black arrow) at a 90° angle to the ulnar axis (Figs. 4 and 5). To ensure consistency, the length of the lever arm was adjusted to equalize the distance and ratio between the load cell’s movement and the fracture displacement. For the sake of simplicity in measurement, the movement of the load cell was approximated as an indication of micromotion during the test. Fixation loss was defined as either 2 mm or more in the load cell’s movement or the occurrence of a catastrophic failure.

The cyclic loading test aimed to measure the stability with an active range of motion (AROM) exercise. To simulate the AROM of the elbow, a force of 5–110 N was applied 500 times to the specimen at a frequency of 1 Hz [13, 14]. The load cell’s moving distance is considered the micromotion during the AROM exercise [12]. The most posterior point of the ulnar cortex served as the reference for displacement measurement. Comparisons were made between baseline fixation and the measurement taken after 500 cycles. Displacement was estimated by digital calipers (Mitutoyo, Neuss, Germany).

For specimens that successfully completed cyclic loading testing without failure, a load-to-failure test was conducted to determine the maximum load at which fixation loss occurred. The load was incrementally increased at a rate of 5 mm/min starting from 0 N.

### Statistical analysis

A power analysis was conducted to determine the appropriate sample size using G*power 3.1 software with reference to a previous study [12]. The required sample size was eight elbows per group to provide 95% power (alpha = 0.05) for the maximum load in the load-to-failure test [12]. Owing to the small number of specimens, the data were statistically assessed using nonparametric analysis. The Mann–Whitney U test was conducted to assess significant differences between Groups 1 and 2. To detect any differences among Groups 2, 3, and 4, a Kruskal–Wallis test was employed as a global test. If the Kruskal–Wallis test revealed significant differences among the groups, a subsequent Mann–Whitney U test was conducted to identify specific significant differences among groups. For post hoc tests, the Bonferroni correction was applied to adjust for multiple comparisons. A p-value < 0.05 was considered statistically significant when comparing Groups 1 and 2. Statistical significance was adjusted using the Bonferroni correction when comparing Groups 2, 3, and 4 (*p* < 0.017, i.e., 0.05, divided by three as the number of tests).

## Results

### Cyclic loading test

**Fig. 6:**
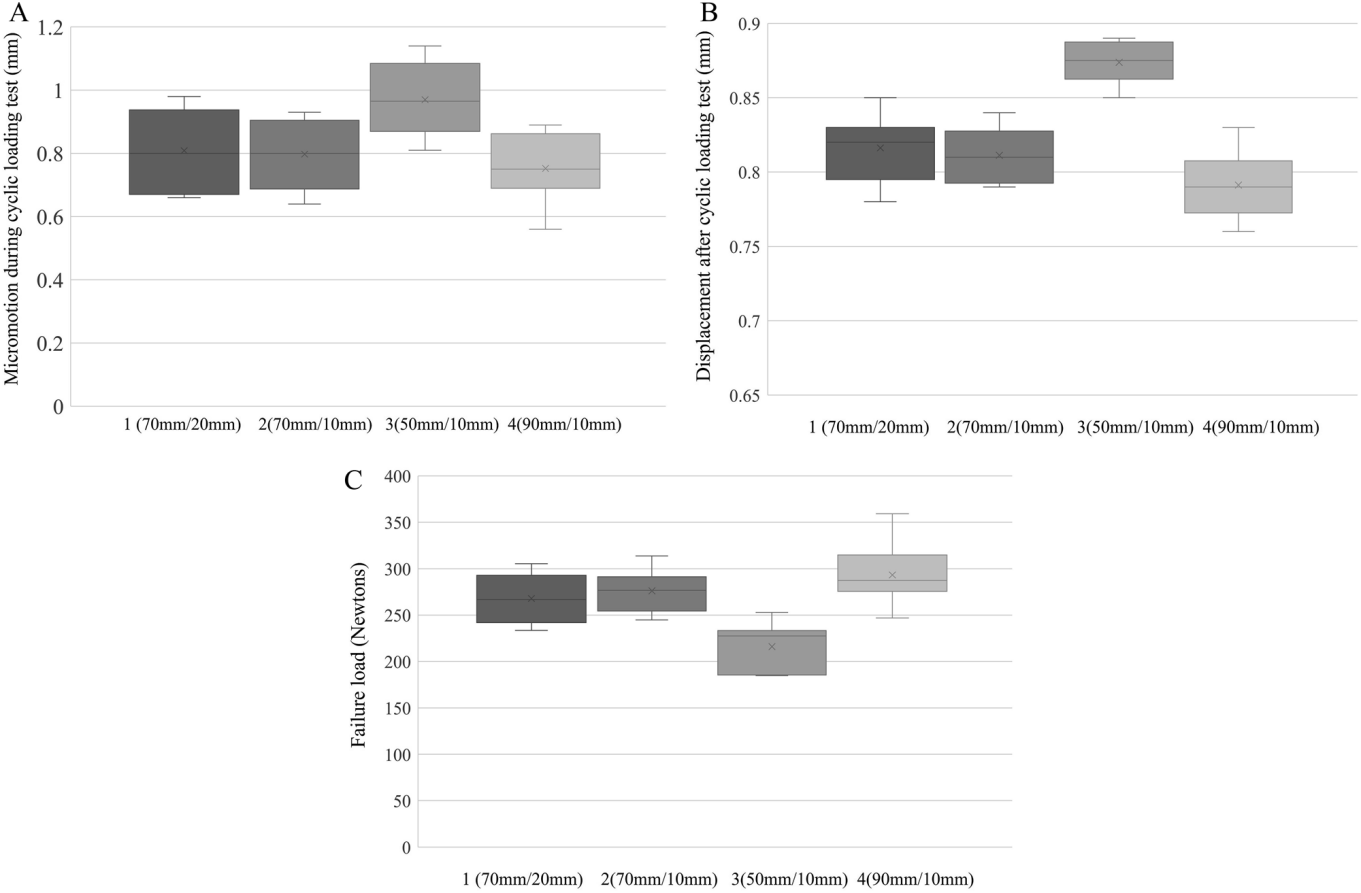
Comparison of biomechanical test among four groups. (**A**) Comparison of mean micromotion during cyclic loading test among four groups. (**B**) Comparison of mean displacement after cyclic loading test among four groups. (**C**) Comparison of failure load among four groups

All groups were stable with a micromotion of < 1.0 mm except for Group 3 (length: 50 mm, interval: 10 mm). There were no significant differences in the mean micromotion and displacement after exercise between Groups 1 (length: 70 mm, interval: 20 mm) and 2 (length: 70 mm, interval: 10 mm) (Table 1). The mean micromotion during the cyclic loading test of Group 3 (length: 50 mm, interval: 10 mm) was significantly higher than that of Groups 2 (length: 70 mm, interval: 10 mm) and 4 (length: 90 mm, interval: 10 mm), although there were no significant differences between Groups 2 and 4 (Fig. 6A). The mean displacement after the cyclic loading test of Group 3 (length: 50 mm, interval: 10 mm) was significantly higher than that of Groups 2 (length: 70 mm, interval: 10 mm) and 4 (length: 90 mm, interval: 10 mm), although there were no significant differences between Groups 2 and 4 (Fig. 6B) (Table 1).

**Table 1:** Mean micromotion and displacement in the Cyclic loading test

Group (length/interval)	Mean micromotion during the test (mm) (range)	Displacement after the test (mm) (range)
1 (70 mm/20 mm)	0.81 ± 0.13 (0.66–0.98)	0.82 ± 0.02 (0.78–0.85)
2 (70 mm/10 mm)	0.80 ± 0.11 (0.64–0.93)	0.81 ± 0.02 (0.79–0.84)
3 (50 mm/10 mm)	0.97 ± 0.12 (0.81–1.14)	0.87 ± 0.01 (0.85–0.89)
4 (90 mm/10 mm)	0.75 ± 0.11 (0.56–0.89)	0.79 ± 0.02 (0.76–0.83)
**P-value**		
Group 1 vs. 2	0.798	0.645
Group 2 vs. 3	0.010*	< 0.001*
Group 3 vs. 4	0.003*	< 0.001*
Group 2 vs. 4	0.442	0.083

Groups 1 and 2 were compared using the Mann–Whitney U test. Given that the Kruskal–Wallis test revealed significant differences between Groups 2, 3, and 4, the Mann–Whitney U test was performed to detect significant differences among the groups. Statistical significance was set at *p* < 0.05 when comparing Groups 1 and 2 and *p* < 0.017 when comparing Groups 2, 3, and 4 after adjusting for the Bonferroni correction as a post hoc test. Values are presented as mean ± standard deviation

* Statistically significant

### Load-to-failure test

**Fig. 7:**
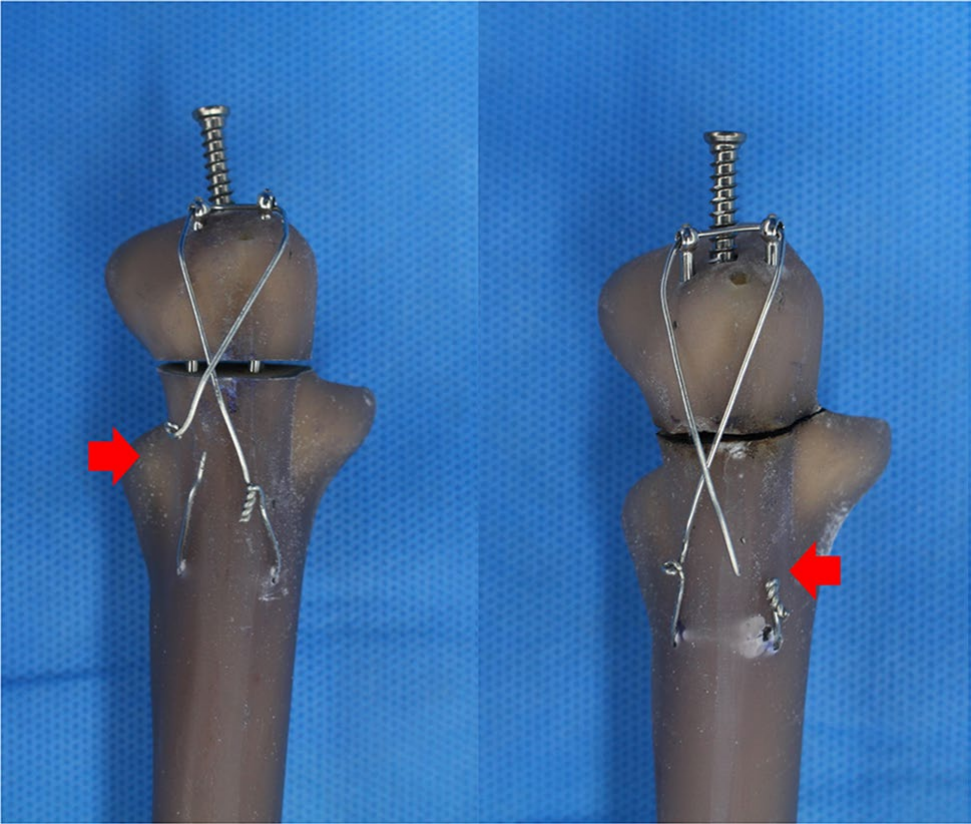
All failures occurred as wire loosening or breakage without cases of ring pin breakage or backing out. All wire breakages occurred at the site of wire knots (red arrows) and not at the ring for wire passage

No significant difference was observed in the maximal load-to-failure test between Groups 1 and 2. The maximal load to failure of Group 3 was significantly lower than that of Groups 2 and 4, although there was no significant difference between Groups 2 and 4 (Fig. 6C) (Table 2). All failures occurred owing to wire loosening or breakage (Fig. 7). There were no cases of load screw or ring-pin breakage.

**Table 2:** Mean maximum load in the load-to-failure test

Group (length/interval)	Maximum load (*N*) (range)
1 (70 mm/20 mm)	267.88 ± 26.11 (233.46–305.3)
2 (70 mm/10 mm)	276.01 ± 22.55 (244.69–313.66)
3 (50 mm/10 mm)	216.06 ± 26.70 (184.71–252.87)
4 (90 mm/10 mm)	293.25 ± 33.98 (246.82–359.31)
**P-value**	
Group 1 vs. 2	0.505
Group 2 vs. 3	0.001*
Group 3 vs. 4	< 0.001*
Group 2 vs. 4	0.279

Groups 1 and 2 were compared using the Mann–Whitney U test. Given that the Kruskal–Wallis test revealed significant differences between Groups 2,3 and 4, the Mann–Whitney U test was performed to detect significant differences among the groups. Statistical significance was set at *p* < 0.05 when comparing Groups 1 and 2 and *p* < 0.017 when comparing Groups 2, 3, and 4 after adjusting for the Bonferroni correction as a post hoc test. Values are presented as mean ± standard deviation

* Statistically significant

## Discussion

This biomechanical study investigated effects of ring-pin length and interval in TBW with ring pins for the treatment of transverse olecranon fractures. Our results demonstrated that the ring-pin length significantly influenced the biomechanical stability of the construct, with 50-mm pins showing inferior fixation strength compared to 70-mm and 90-mm pins. In contrast, pin intervals (10 mm vs. 20 mm) did not result in a significant difference in micromotion or maximal load to failure. Furthermore, all observed failures were due to wire loosening or breakage, and not pin migration or breakage, supporting the mechanical stability of ring pins and their effectiveness in minimizing complications related to pin migration.

Displaced olecranon fractures should be treated surgically with open reduction and internal fixation using plates, intramedullary nails, and TBW [1, 2]. Although TBW is commonly used for simple transverse olecranon fractures, it is associated with several complications. One of the main complications observed after conventional TBW is backing out of the K-wires, leading to a higher reoperation rate, particularly for implant removal [15]. To address this issue, a modified TBW technique was proposed that involves oblique placement of the K-wire from proximal to distal to capture the anterior ulnar cortex, improve stability, and reduce the risk of the K-wire backing out [16, 17]. However, despite this modification, the distal tip of the K-wire has been associated with other postoperative complications, such as adjacent vessel pseudoaneurysm or impairment of forearm motion [18, 19].

To overcome these problems, several researchers treated these fractures with TBW using ring pins and reported favorable clinical outcomes [6, 7, 9, 20]. Ring pins were inserted along the intramedullary space to eliminate postoperative complications associated with anterior transcortical fixation. Moreover, after passing through the rings and ulna, the cerclage wire was twisted to form a single construct consisting of a wire, ring pins, and ulna. This configuration effectively prevents proximal migration of the ring pin unless the wire breaks, thus minimizing the backing out of ring pins. Kim et al. [6] successfully treated 44 olecranon fractures with ring pins and observed no pin migration or loss of reduction. Okamoto et al. [7] treated 24 olecranon fractures without encountering any cases of pin migration. Shimura et al. [9] compared the efficacy of TBW with ring pins to anatomical locking plates in 58 patients. The locking plate group exhibited a higher incidence of complications than the ring-pin group despite comparable clinical outcomes. Moreover, Sadri et al. [8] conducted a biomechanical study and determined that TBW using ring pins could offer comparable stability to AO-modified TBW, which involves the placement of K-wires penetrating the anterior cortex of the ulna.

However, there have been several variations in the length of the ring pins among studies, and few studies have described the interval between the two ring pins. Sadri et al. [8] and Shimura et al. [9] used 80-mm ring pins, Kim et al. [6] suggested 90-mm ring pins, and Takada et al. [20] inserted 70-mm ring pins. We used 70-mm or 90-mm ring pins, depending on the patient’s size. Although they did not explicitly describe the interval between pins, it can be seen from the presented figures that most of the authors inserted ring pins in parallel or in a slightly converging direction.

Therefore, we aimed to determine the optimal insertion interval and length of ring pins for TBW with ring-pin systems in the treatment of transverse olecranon fractures. Based on biomechanical testing, we found that the insertion interval of the ring pins did not significantly affect the strength of the TBW construct. Although we expected that a wider pin interval would affect biomechanical strength by allowing the wire to distribute tension over a broader area, our findings did not support this assumption. As such, we recommend inserting two ring pins in parallel at 10-mm intervals, as further widening of the interval provides no biomechanical benefit and may result in technical difficulties due to the anatomical features of the ulna, where the diameter of the medullary cavity narrows distally.

Moreover, using a ring pin with a length of 50 mm resulted in weaker biomechanical stability. In both cyclic loading and load-to-failure tests, the group using 50-mm ring pins showed significantly lower strength compared to the groups using 70-mm and 90-mm ring pins, although no significant differences were observed between the groups using 70-mm and 90-mm ring pins. Therefore, we recommend the use of ring pins with a length of at least 70 mm for TBW in treating transverse olecranon fractures. As shown in the results of our biomechanical study, if the TBW procedure is appropriately applied using ring pins of 70 mm or more, it is possible to obtain sufficient stability to initiate early active elbow range of motion exercise.

In this study, all failures occurred because of wire loosening or breakage without ring-pin breakage or backing out, suggesting that ring pins offer reliable mechanical stability and may help to reduce the risk of complication associated with pin migration (Fig. 7). Sadri et al. [8] suggested that the friction between the ring and wire could increase the risk of wire breakage, but all wire breakages occurred at the site of the wire knots and not at the ring for wire passage in our study (Fig. 7).

This study has several limitations. First, the tension of the wire knots was not standardized during the TBW procedure. To minimize variability, all procedures were performed by a trauma surgeon with > 20 years of experience and with extensive experience in TBW using ring pins [6, 21]. Second, our method for stimulating physiologic muscle interaction differed from those used in previous biomechanical studies [22, 23]. Our approach was based on validated protocols from a previous study using the same bone model [12], which suggests that direct screw loading reduces variability by eliminating interference from polyester bands or steel wires. The relatively consistent results obtained, with a limited standard deviation and no catastrophic failure due to load-screw breakage, suggest that our experimental setup was appropriate. Third, although fourth-generation composite bones mimic the structural characteristics of human bone, they lack surrounding soft tissues such as muscles and tendons, which limits the generalizability of our results to clinical practice. Therefore, the findings should be viewed as indicative of biomechanical trends rather than as definitive clinical outcomes. Finally, the small sample size reduced the statistical power of this study. Future research incorporating finite element analysis and prospective clinical trials is necessary to validate and extend these findings.

## Conclusions

Based on the biomechanical tests, we recommend inserting two ring pins in parallel at a 10-mm interval and with a length of at least 70 mm for TBW in transverse olecranon fractures. Further widening of the pin interval provides no biomechanical benefit and may result in technical difficulties owing to the anatomical features of the ulna, and 50-mm ring pins show significantly lower mechanical strength.

## Data Availability

The datasets used and/or analysed during the current study are available from the corresponding author on reasonable request.

## Abbreviations

AROM: Active Range Of Motion
TBW: Tension Band Wiring

## References

[1] Siebenlist S, Buchholz A, Braun KF. Fractures of the proximal ulna: current concepts in surgical management. EFORT Open Rev. 2019;4:1–9. 30800474 10.1302/2058-5241.4.180022 PMC6362340

[2] Baecher N, Edwards S. Olecranon fractures. J Hand Surg Am. 2013;38:593–604. 23428192 10.1016/j.jhsa.2012.12.036

[3] Chalidis BE, Sachinis NC, Samoladas EP, Dimitriou CG, Pournaras JD. Is tension band wiring technique the gold standard for the treatment of olecranon fractures? A long term functional outcome study. J Orthop Surg Res. 2008;3:9. 18294381 10.1186/1749-799X-3-9 PMC2265682

[4] Saeed ZM, Trickett RW, Yewlett AD, Matthews TJ. Factors influencing K-wire migration in tension-band wiring of olecranon fractures. J Shoulder Elb Surg. 2014;23:1181–6. 10.1016/j.jse.2014.02.018 24875733

[5] Schneider MM, Nowak TE, Bastian L, Katthagen JC, Isenberg J, Rommens PM, et al. Tension band wiring in olecranon fractures: the myth of technical simplicity and osteosynthetical perfection. Int Orthop. 2014;38:847–55. 24326359 10.1007/s00264-013-2208-7 PMC3971280

[6] Kim JY, Lee YH, Gong HS, Lee SL, Lee SK, Baek GH. Use of Kirschner wires with eyelets for tension band wiring of olecranon fractures. J Hand Surg Am. 2013;38:1762–7. 23849734 10.1016/j.jhsa.2013.05.012

[7] Okamoto M, Namba J, Kuriyama K, Miyamura S, Yokoi H, Yamamoto K. Surgical technique in tension band wiring method for selected comminuted olecranon fractures. Eur J Orthop Surg Traumatol. 2020;30:237–42. 31538271 10.1007/s00590-019-02551-y

[8] Sadri H, Stern R, Singh M, Linke B, Hoffmeyer P, Schwieger K. Transverse fractures of the olecranon: a Biomechanical comparison of three fixation techniques. Arch Orthop Trauma Surg. 2011;131:131–8. 20680308 10.1007/s00402-010-1156-6

[9] Shimura H, Nimura A, Fujita K, Kaburagi H. Comparison of the efficacy of the tension band wiring with eyelet wire versus anatomical locking plate fixation for the treatment of displaced olecranon fractures. J Orthop Surg (Hong Kong). 2021;29:23094990211059231. 34872400 10.1177/23094990211059231

[10] Heiner AD. Structural properties of fourth-generation composite femurs and tibias. J Biomech. 2008;41:3282–4. 18829031 10.1016/j.jbiomech.2008.08.013

[11] Jones TB, Karenz AR, Weinhold PS, Dahners LE. Transcortical screw fixation of the olecranon shows equivalent strength and improved stability compared with tension band fixation. J Orthop Trauma. 2014;28:137–42. 23681413 10.1097/BOT.0b013e31829a25d2

[12] Lee Y, Cho BW, Kim MB, Lee YH. Biomechanical comparison between double-plate fixation and posterior plate fixation for comminuted olecranon fracture using two triceps screws in synthetic bone model. Medicine. 2022;101:e28313. 35029878 10.1097/MD.0000000000028313 PMC8735719

[13] Prayson MJ, Williams JL, Marshall MP, Scilaris TA, Lingenfelter EJ. Biomechanical comparison of fixation methods in transverse olecranon fractures: a cadaveric study. J Orthop Trauma. 1997;11:565–72. 9415862 10.1097/00005131-199711000-00004

[14] Midtgaard KS, Søreide E, Brattgjerd JE, Moatshe G, Madsen JE, Flugsrud GB. Biomechanical comparison of tension band wiring and plate fixation with locking screws in transverse olecranon fractures. J Shoulder Elb Surg. 2020;29:1242–8. 10.1016/j.jse.2020.01.079 32139286

[15] Romero JM, Miran A, Jensen CH. Complications and re-operation rate after tension-band wiring of olecranon fractures. J Orthop Sci. 2000;5:318–20. 10982677 10.1007/s007760070036

[16] Mullett JH, Shannon F, Noel J, Lawlor G, Lee TC, O’Rourke SK. K-wire position in tension band wiring of the olecranon - a comparison of two techniques. Injury. 2000;31:427–31. 10831740 10.1016/s0020-1383(00)00014-0

[17] van der Linden SC, van Kampen A, Jaarsma RL. K-wire position in tension-band wiring technique affects stability of wires and long-term outcome in surgical treatment of olecranon fractures. J Shoulder Elb Surg. 2012;21:405–11. 10.1016/j.jse.2011.07.022 22036542

[18] Lee SH, Han SB, Jeong WK, Park JH, Park SY, Patil S. Ulnar artery pseudoaneurysm after tension band wiring of an olecranon fracture resulting in volkmann’s ischemic contracture: a case report. J Shoulder Elb Surg. 2010;19:e6–8. 10.1016/j.jse.2009.06.007 19740681

[19] Candal-Couto JJ, Williams JR, Sanderson PL. Impaired forearm rotation after tension-band-wiring fixation of olecranon fractures: evaluation of the transcortical K-wire technique. J Orthop Trauma. 2005;19:480–2. 16056081 10.1097/01.bot.0000164338.79013.10

[20] Takada N, Kato K, Fukuta M, Wada I, Otsuka T. Minimally invasive tension band wiring technique for olecranon fractures. Tech Hand Up Extrem Surg. 2013;17:199–201. 24240623 10.1097/BTH.0b013e3182a9128c

[21] Lee SH, Kim MB, Lee YH. Simple osteotomy site repair method for the trans-olecranon approach: tension band wiring with ring pins. J Hand Surg Asian Pac Vol. 2021;26:571–9. 34789092 10.1142/S2424835521500545

[22] Hammond J, Ruland R, Hogan C, Rose D, Belkoff S. Biomechanical analysis of a transverse olecranon fracture model using tension band wiring. J Hand Surg Am. 2012;37:2506–11. 22995702 10.1016/j.jhsa.2012.07.025

[23] Moon M, Schweser K, Bezold W, Cook JL. Biomechanical comparison of continuous compression implants versus tension band fixation for transverse olecranon fractures. J Orthop. 2022;34:316–21. 36204515 10.1016/j.jor.2022.09.015 PMC9531040

